# A Feasibility Study of the WHO Digital Mental Health Intervention Step-by-Step to Address Depression Among Chinese Young Adults

**DOI:** 10.3389/fpsyt.2021.812667

**Published:** 2022-01-07

**Authors:** Hao Fong Sit, Ieng Wai Hong, Sebastian Burchert, Elvo Kuai Long Sou, Mek Wong, Wen Chen, Agnes Iok Fong Lam, Brian J. Hall

**Affiliations:** ^1^Department of Psychology, The University of Hong Kong, Pokfulam, Hong Kong SAR, China; ^2^Moon Chun Memorial College, University of Macau, Macau, Macau SAR, China; ^3^Department of Education and Psychology, Division of Clinical Psychological Intervention, Freie Universität Berlin, Berlin, Germany; ^4^Student Affairs Office, University of Macau, Macau, Macau SAR, China; ^5^Department of Medical Statistics, School of Public Health, Sun Yat-sen University, Guangzhou, China; ^6^Centre for Macau Studies, University of Macau, Macau, Macau SAR, China; ^7^Center for Global Health Equity, NYU Shanghai, Shanghai, China; ^8^Department of Health, Behavior, and Society, Johns Hopkins Bloomberg School of Public Health, Baltimore, MD, United States

**Keywords:** depression, digital health, e-mental health, minimally guided intervention, feasibility, Chinese young adults

## Abstract

**Background:** Chinese young adults experience barriers to mental health treatment, including the lack of treatment providers and stigma around treatment seeking. Evidence-based digital mental health interventions are promising and scalable alternatives to face-to-face treatment for this population, but lack rigorous evidence to support scale-up in China.

**Aim:** The study was a feasibility study for a large-scale RCT of Step-by-Step, a behavioral activation-based, mental health intervention to address depression and anxiety symptoms in Chinese young adults. It sought to assess feasibility of recruitment and of delivery of Step-by-Step in a University setting, to assess acceptability of the intervention, and to examine potential effectiveness.

**Method:** An uncontrolled, feasibility trial was conducted to assess the feasibility and acceptability of Chinese Step-by-Step for Chinese University students with elevated depressive symptoms (PHQ-9 scores at or above 10) in Macao, China. Data was collected at two different time points (i.e., baseline and 8-weeks after baseline), administered via questionnaires embedded in an interventional mobile application. Participation rate and dropout rate were measured. Depressive and anxiety symptom severity, well-being, and self-defined stress were assessed. Satisfaction with the program was assessed using qualitative interviews.

**Results:** A total of 173 students were screened, 22.0% (*n* = 38) were eligible, and 63.2% of them (*n* = 24) started the intervention. The dropout rate by post-test was 45.8%. Results from completers showed that Step-by-Step was potentially effective in reducing depressive and anxiety symptom severity, and self-defined stress. Students were generally satisfied with the program, but also offered suggestions for continued improvement. Qualitative feedback was reported within the RE-AIM framework, covering recruitment, effectiveness, adoption, implementation, and maintenance. Amendments to the program were made according to the feedback (e.g., adding notification for new session, modify the time zone).

**Conclusion:** A minimally guided Step-by-Step protocol and the study procedure were successfully pilot tested for use for Chinese University students. The intervention was acceptable and no adverse events were reported. The results support the potential effectiveness and feasibility of a large-scale evaluation of the program.

## Introduction

Evidence-based digital mental health interventions have gained empirical support and their efficacy is widely supported (1–4). This novel delivery model can reduce the treatment gap (5) and stigma-related barriers to seeking mental health treatment (6, 7), but few rigorous studies on digital mental health interventions were conducted within low- and middle-income countries (LMIC) (8), and among Chinese people in particular. Evidence from a meta-analysis of 22 randomized controlled trials including 4,104 participants showed that digital mental health interventions were effective in alleviating symptoms of depression, anxiety, and post-traumatic stress disorder, with a moderate to large effect size (8). In the past decade, there was an increase in digital mental health interventions for Chinese, but few were developed or adapted for depression, and even fewer addressed depression through smartphone applications, according to a recent systematic review on digital mental health in mainland China (9).

Emerging adulthood is a crucial developmental stage when individuals are undergoing complex and dynamic changes (10), with increased vulnerability to poor mental health because of the developmental, academic, occupational, and social challenges they encounter (10, 11). University students, a large community of young adults, are at risk of a variety of mental health issues, and depression and anxiety are common in this population (11–15). In China, roughly 30% of college students have clinically significant depression (14). Such high prevalence was also witnessed during the COVID-19 pandemic (16), and a shift of delivery model from conventional approaches (e.g., face-to-face) to digital treatment (e.g., through digital devices) has increased the public's awareness. However, evidence for the use of digital mental health interventions for depression in China is still lacking, and the scalability and implementation of these services is uncertain.

To improve access to interventions globally, the World Health Organization (WHO) developed technology-assisted interventions that have the potential to scale (17). In collaboration with the Ministry of Public Health in Lebanon, University of Zurich, Switzerland, and Freie Universität Berlin, Germany, the WHO developed the web version of Step-by-Step (SbS) (17), which was later developed into a mobile app and culturally adapted for a diversity of populations (18–23). In order to evaluate the effectiveness and implementation of SbS in a Chinese tertiary educational setting, SbS was culturally adapted (23), followed by the current feasibility study that preliminarily tested the adapted intervention, and informed the study process of a future randomized controlled trial (RCT), which is now planned (24), guided by the development-evaluation-implementation process (25) and implementation evaluation framework (26, 27).

The objectives of the current study were to assess the feasibility of delivering Step-by-Step in a University setting for Chinese young adults utilizing a minimal peer-support guidance model and obtain preliminary evidence of the effectiveness of the intervention to reduce depressive and anxiety symptom severity.

## Methods

### Trial Design

An uncontrolled, single-arm pilot trial was conducted to understand the feasibility and acceptability of Chinese Step-by-Step for University students with elevated depressive symptoms in Macao (SAR), China. Data were collected at two different time points (i.e., baseline and 8-weeks after baseline), administered *via* questionnaires embedded in the interventional mobile application. Although the current feasibility study adopted an uncontrolled design, it followed the CONSORT statement (28).

### Participants

#### Eligible Criteria

Participants were included if they had a score of Patient Health Questionnaire-9 (PHQ-9) of 10 or higher, were students, at least 18 years old, Chinese nationality, Cantonese or Mandarin-speaking, had a permit to stay in Macao, and had a digital device to access the intervention (e.g., smartphone). Participants were excluded if they reported high levels of suicidal ideation or a plan to self-harm in the past month.

#### Setting and Recruitment

The University of Macau is the largest public University in Macao. It hosts roughly 10,000 local and mainland Chinese students. It has a residential college system where students reside and participate in co-curricular and general education program. Moon Chun Memorial College, one of the 10 residential colleges, houses approximately 500 students, and served as a context for participant recruitment and data collection for this feasibility study. Multiple strategies were adopted for recruitment within the residential college, including posters, mass email invitations, referrals, in-class recruitment, posts in social networking application, and a booth in residential college events.

All eligible participants in the feasibility study were allocated to the 5-session Step-by-Step intervention to test the study procedure, and acceptability of adapted content, and obtain evidence of potential intervention effectiveness. SbS was delivered via a smartphone application using iOS and Android systems or a website that can be accessed on digital devices (e.g., smartphone, laptop).

### Intervention

SbS is a 5-session illustrated narrative program designed to be transdiagnostic with a primary focus on depression, which includes components of psychoeducation, relaxation techniques, identifying personal strengths, positive self-talk, enhancing social support, and relapse prevention, with behavioral activation as the core therapeutic component and based on WHO guidelines and recommendations (17, 29). It is delivered in an 8-week timeframe through digital devices. Before entering the five therapeutic sessions, each user went through an onboarding session in the app, which explained the study, obtained consent, and confirmed eligibility. Consent items were presented one by one. The consent progress only proceeded when the participant chose “agree,” or the process of consent and the program ended if “disagree” were chosen. Eligible participants entered an introduction session (session 0) consisting of an introduction to SbS sessions and a baseline assessment. Each therapeutic session consisted of 2-3 parts with illustrated narratives and exercises and lasted around 20-30 min. Participants were recommended to finish one session per week before a new session was unlocked. Three main types of exercises were provided in the application, including behavioral activation (e.g., activity scheduling), self-care (e.g., gratitude exercise), and relaxation (e.g., breathing exercise). The mood tracking feature allowed users to record their mood every day. Each participant is assigned an e-helper, a trained non-specialist who provides minimal support by phone and text to participants. E-helpers were responsible for check-in with the participants, motivate them to complete one session per week, and solve technique problems concerning using the app on a weekly basis. Each e-helper handled no more than four active users at the same time. After the 8 week of intervention period, regardless whether the five sessions were completed or not, participants were invited to interview, and they could go on learning the remaining sessions if they had not finished within 8 weeks and using the exercises in the app.

#### E-Helper Training

As part of the program, e-helpers provided minimal weekly guidance. In this pilot study, we trained peers to offer this guidance, as this was deemed to be most likely to support scalability and maintenance of the program. E-helpers were unpaid undergraduate peer counselors from our partner residential college, undergraduate research assistants, and a masters-level counseling and psychotherapy postgraduate interns from the Students Affairs Office. E-helper training was provided by the psychological counselors from the University and the study coordinator from the research team. The 24-h training covered mental health first-aid (12 h), an overview of Step-by-Step, clinical training on how to communicate during weekly contacts (9 h), and technical training on the mobile application (3 h), was held during the weekend or weekday evenings to fit the e-helpers' schedule. The sub-set of student peer counselor e-helpers (*n* = 13) who were recruited for this study were evaluated using performance-based role-play exercises before they were deemed appropriate to provide guidance to other students. All e-helpers received weekly 1-h group supervision during the study intervention period.

### Measures

#### Overview

Participant sociodemographic information including age, sex, educational level, marital status, place of birth, identification for staying in Macao and previous psychological or mental health service use (Yes/No), were collected. Feasibility and acceptability of the intervention were evaluated by rate of participation and dropout, and the process of intervention implementation that with a focus on recruitment strategy, data collection tool, and intervention adoption (30).

#### Primary Outcome Measure

The Chinese version of the self-reported Patient Health Questionnaire-9 (PHQ-9) (31), was used to measure depression symptom severity with a four-point scale, ranging from 0 (*not at all*) to 3 (*nearly every day*) and higher scores indicating higher depressive symptoms (0-27). A cut-off score of 10 was used to detect the clinical presence of depression in our sample (32, 33). The Chinese PHQ-9 was validated and showed excellent internal consistency in the Chinese population (α > 0.80) (32–34).

#### Secondary Outcome Measures

The Chinese version of the General Anxiety Disorder-7 (GAD-7) (35–37) and World Health Organization-Five Well-Being Index (WHO-5) (38, 39) were used to evaluate anxiety and well-being and demonstrated good reliability (αs > 0.80) and validity in Chinese populations. The Psychological Outcome Profiles (PSYCHOLOPS) (40) was used to evaluate client-defined stress and was translated from the original English version by a Chinese-English bilingual research assistant, and a backward translation was done by another Chinese-English research assistant to ensure the items are cultural and conceptual equivalent in the study.

#### Implementation Evaluation

A qualitative interview guide was adapted from another digital mental health project (21) and further modified based on the RE-AIM framework (26, 27). The RE-AIM framework has 5 dimensions, including recruitment (“*How do I reach the targeted population with the intervention?*”), efficacy or effectiveness (“*How do I know the intervention is efficacious or effective?*”), adoption (“*How do I develop organizational support to deliver the intervention?*”), implementation (“*How do I ensure the intervention is delivered properly?*”), and maintenance (“*How do I incorporate the intervention for its long term delivery?*”) (26, 27). The interview guide was used to evaluate the process of the study covering the following aspects: recruitment, the effect of the intervention, implementation, adoption, and maintenance (Appendix 1 in Supplementary Material). Since Step-by-Step was a minimal-guided, digital, self-help intervention, participants were considered as the major agent regarding intervention adoption.

#### Dropout and Adherence

A record of dropout, reason of dropout, and a number of completed sessions were recorded throughout the study. Reasons of dropout were obtained from the weekly contact with the participant by e-helpers.

### Analysis

#### Statistical Analysis

Descriptive statistics (mean and standard deviation for continuous variables and frequency and proportions for categorical variables) described participants' demographic, clinical characteristics, and treatment adherence. Treatment outcomes were graphically summarized across time points. Paired sample *t*-tests were performed to obtain within-group change in study outcomes from baseline to post-test assessment. For the purpose of our analyses and comparison with previous studies (20, 41), we considered completers to be anyone who at least partially completed session 0 and 3 additional sessions, starters to be anyone who at least partially completed session 0 and completed 1-2 additional sessions, and non-starters to be anyone who completed none of the interventional sessions (i.e., session 1-5). Both starters and non-starters who completed session 0 were considered as dropouts. We used Stata 16 for Windows (42) for the analysis of the data.

#### Qualitative Analysis

Content analysis was applied to identify and describe the five dimensions of the RE-AIM framework (26, 27). This framework is applied to understand key dimensions for the implementation of evidence-based interventions. We adopted this framework to allow us to gather additional information on the long-term maintenance and scalability of the program following a randomized controlled trial. All interviews were transcribed verbatim and translated from Chinese to English, and two assistants coded each transcript independently. The two coders reached a consensus on the understanding of each code by discussion and invited a third coder to decide the code for the context when consensus could not be reached.

### Monitoring Harms

Harm is defined as a sustained deterioration directly caused by the intervention (43). The PHQ-9 was used to monitor participants' depressive symptoms weekly, which informed e-helpers about the support that needed to be offered. In addition, if any adverse event (e.g., self-harm, symptom deterioration, distress) occurred, the e-helper would report to the clinical supervisor of the e-helper management team. A referral would be made for participants who reported any life-threatening event (i.e., self-harm, suicidal attempt) to the University counseling center where a professional team of social workers, psychological counselors, and psychotherapists could provide support, including referrals to additional services.

### Ethics and Consent

The study obtained ethics approval at the University of Macau (MYRG2018-00241-FSS), and all participants provided consent to participate in the study.

## Results

### Sample Descriptions

**Figure 1:**
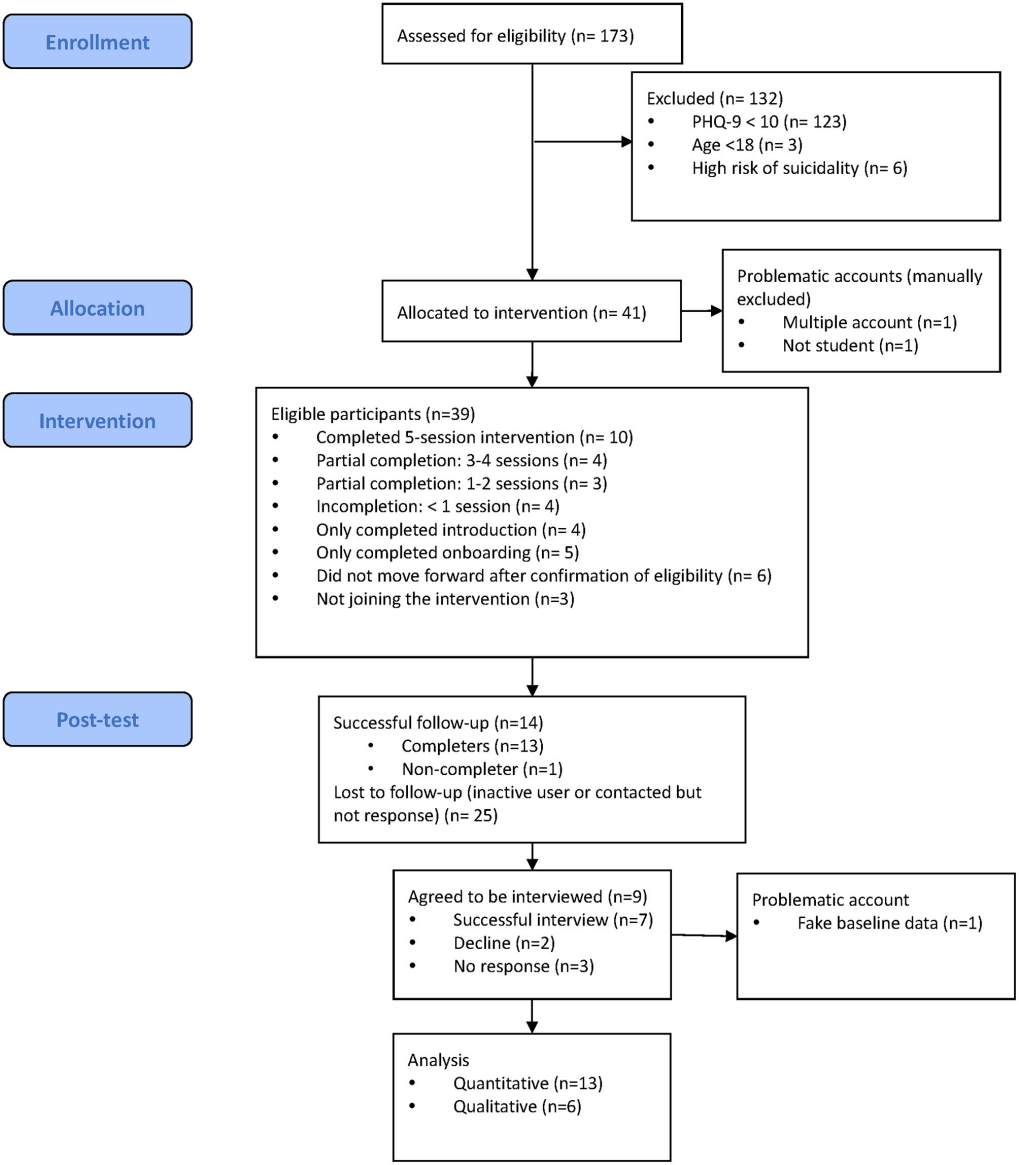
Study overview.

Details of the study overview are shown in Figure 1. A total of 173 participants were assessed for eligibility between October 2020 and November 2020. One hundred and thirty-two participants (76.3%) were excluded because of low levels of depressive symptoms (*n* = 123, 71.1%), aged below 18 (*n* = 3, 1.7%), and high risk of suicidality (*n* = 6, 3.5%). Among the excluded participants, most of them were female (*n* = 68, 54.5%), and local students (*n* = 76, 57.6%).

**Figure 2:**
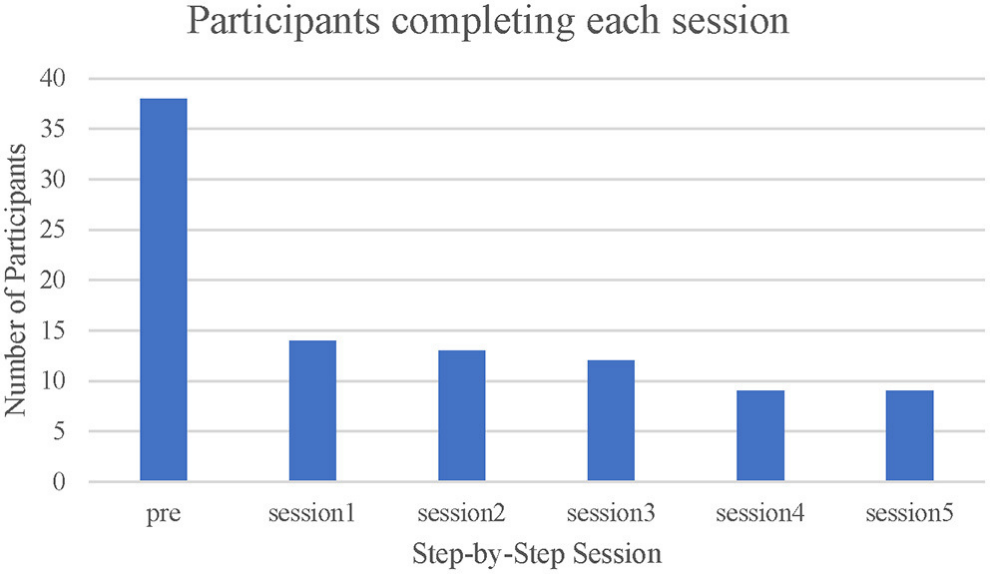
Number of participants completing each session.

Forty-one users were eligible. Among them, three accounts were removed (one account was not functional, one was not a student, and one was removed during analysis as it was discovered they had reported high symptoms to enter the trial). Figure 1 showed the participant flow. Figure 2 showed the number of participants who completed each session. A total of 38 eligible participants were included, 14 did not start any session of SbS (5 only answered the baseline survey [on boarding session], 6 did not try any part of a session after confirmation of eligibility, and 3 self-excluded). Among the 24 participants who started SbS, 4 only completed the introduction session [session 0], 4 tried some parts of session 1, 3 completed 1-2 sessions, 4 completed 3-4 sessions (including one participant who completed 2 of the 3 parts in session 3), and 9 completed the 5-session intervention. Based on our definition of non-starter (see section Statistical Analysis), 15 non-starters were identified, including 5 only completing the onboarding session, 6 trying no part of a session after confirmation of eligibility, and 4 only completing the introduction session (session 0). Among those who started any session of SbS (including session 0), 11 participants who completed fewer than 3 sessions (45.8%) were considered dropouts and 13 participants (54.2%) were considered completers (completed at least three therapeutic sessions). Reasons for drop-out included insufficient time due a busy academic timetable, joining the program only for e-helper support, and not being aware that they needed to complete the intervention in 8 weeks. One completer did not complete the post-test questionnaire. No self-harm, symptom deterioration, increased distress, or suicidal attempt was reported. Details of their demographic characteristics and study outcomes are shown in Tables 1, 2.

**Table 1:** Participant characteristics at baseline (*N* = 38).

	**Completers (*n* = 13)**	**Starters (*n* = 7)**	**Non-starters (*n* = 15)**	**Self-excluded (*n* = 3)**
	***n* (%)**	***n* (%)**	***n* (%)**	***n* (%)**
Age (18-25, M, *SD*)	19.00 (0.91)	20.14 (2.19)	18.46 (0.66)	18 (0)
**Gender**				
Male	3 (7.9%)	3 (7.9%)	2 (5.3%)	3 (7.9%)
Female	10 (26.3%)	4 (10.5%)	12 (31.6%)	-
**Education**				
Year 1	7 (20.5%)	2 (5.3%)	11 (28.9%)	1 (2.6%)
Year 2	3 (7.9%)	2 (5.3%)	1 (2.6%)	-
Year 3	2 (5.3%)	1 (2.6%)	1 (2.6%)	-
Year 4	1 (2.6%)	2 (5.3%)	-	-
**Born in Macau**				
Yes	6 (15.8%)	2 (5.3%)	5 (13.2%)	1 (2.6%)
No	7 (18.4%)	5 (13.2%)	8 (21.1%)	-
**Macao ID holder**				
Yes	11 (28.9%)	3 (7.9%)	8 (21.1%)	1 (2.6%)
No	2 (7.7%)	4 (10.5%)	5 (13.2%)	-
**From where to know about SbS**				
Residential fellow (RF)	10 (26.3%)	7 (18.4%)	9 (23.7%)	1 (2.6%)
Student counsellor/social worker	1 (2.6%)	-	-	-
Friend	-	-	2 (5.3%)	-
Organization/NGO	1 (2.6%)	-	1 (2.6%)	-
Others	1 (2.6%)	-	1 (2.6%)	-
**Mental health service in the last 3 months (baseline)**				
Yes	1 (2.6%)	3 (7.9%)	1 (2.6%)	-
No	12 (31.6%)	4 (10.5%)	11 (28.9%)	1 (2.6%)
**Mental disorders diagnosis in the last three months (baseline)**				
Yes	-	-	-	
No	13 (34.2%)	7 (18.4%)	12 (31.6%)	1 (2.6%)
**Mental disorders medication in the last 3 months (baseline)**				
Yes	1 (2.6%)	-	-	-
No	12 (31.6%)	7 (18.4%)	12 (31.6%)	1 (2.6%)
**Psychotherapy currently/in the past 3 months**				
Yes	1 (2.6%)	1 (2.6%)	-	-
No	12 (31.6%)	6 (15.4%)	12 (31.6%)	1 (2.6%)

**Table 2:** Study outcome variables at baseline (*N* = 38).

	**Completers (*n* = 13)**		**Starters (*n* = 7)**		**Non-starters (*n* = 15)**		**Self-excluded (*n* = 3)**	
	**M**	**SD**	**M**	**SD**	**M**	**SD**	**M**	**SD**
PHQ-9	13.07	2.93	12.86	4.18	13.8	3.14	11	1
GAD-7	9.85	4.08	10.00	4.16	10.55	4.41	14	.
WHO-5	11.77	6.60	9.57	4.65	10.73	4.58	9	.
PSYCHOLOPS	12.31	5.19	13.00	2.71	13.00	2.00	15	.

*PHQ-9, The Patient Health Questionnaire-9; GAD-7, The General Anxiety Disorder-7; WHO-5, The World Health Organization- Five Well-Being Index; PSYCHLOPS,The Psychological Outcome Profiles. In Non-starters, 11 participants completed GAD-7 and WHO-5, and 7 completed PSYCHOLOPS. Among those who self-excluded, 1 participant completed GAD-7, WHO-5 and PSYCHOLOPS*.

For completer sub-group analyses, the 8 of the 13 participants who answered the post-test assessment completed the intervention were categorized as full completers (*n* = 8), and 4 who did some sessions as partial completers (*n* = 4), and 1 person who did not complete any interventional session were considered as a control, for non-statistical comparison.

### Primary Outcomes

Twelve participants who did the intervention and completed post-intervention assessment were included in the completer analyses. Mean, *SD*, correlation coefficient (*r*), and effect size (Cohen's *d*) for PHQ-9, GAD-7, WHO-5, and PSYCHOLOPS are shown in Table 3.

**Table 3:** Baseline and post-test changes among completers.

	**Baseline**		**Post-test**		***T*** **-test**			***r***	**Cohen's *d***
	**M**	**SD**	**M**	**SD**	**t**	**df**	***p***		
PHQ-9	13.08	3.06	5.83	5.84	4.29	11	0.001^**^	0.26	1.24
GAD-7	9.75	4.25	6.75	3.82	2.61	11	0.024^*^	0.52	0.75
WHO-5	12.5	6.32	14.25	5.77	1.34	11	0.208	0.72^**^	0.39
PSYCHOLOPS	12	5.29	9.17	4.02	3.44	11	0.006^**^	0.85^**^	0.99

*N = 12 (One completer did not answer post-test). PHQ-9, The Patient Health Questionnaire−9; GAD-7, The General Anxiety Disorder-7; WHO-5, The World Health Organization-Five Well-Being Index; PSYCHLOPS, the Psychological Outcome Profiles.*

* *p < 0.05,*

** *p < 0.01. Cohen's d: 0.2 (small), 0.5 (medium), 0.8 (large)*.

**Figure 3:**
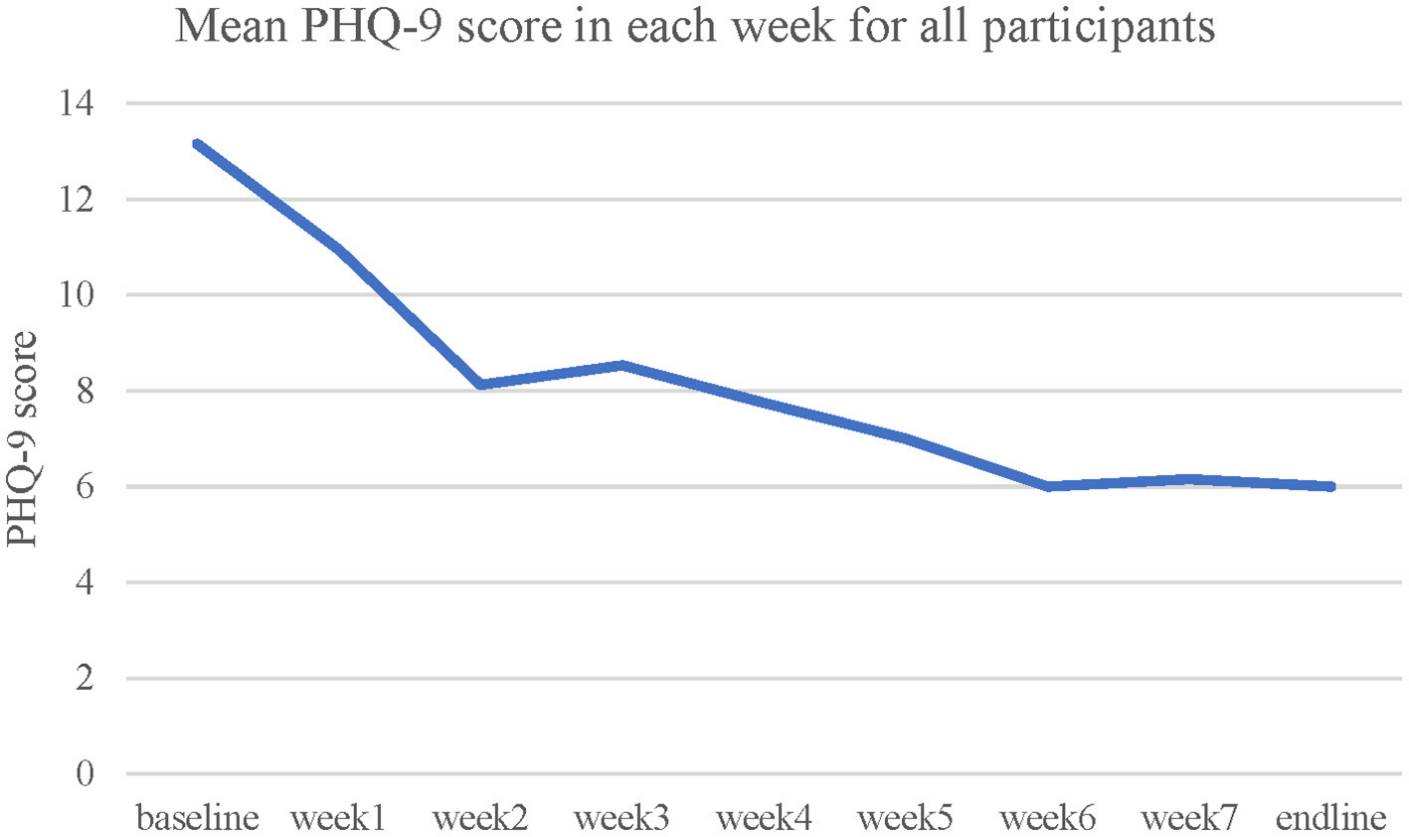
Mean PHQ-9 score in each week for all participants across 8 weeks.

**Figure 4:**
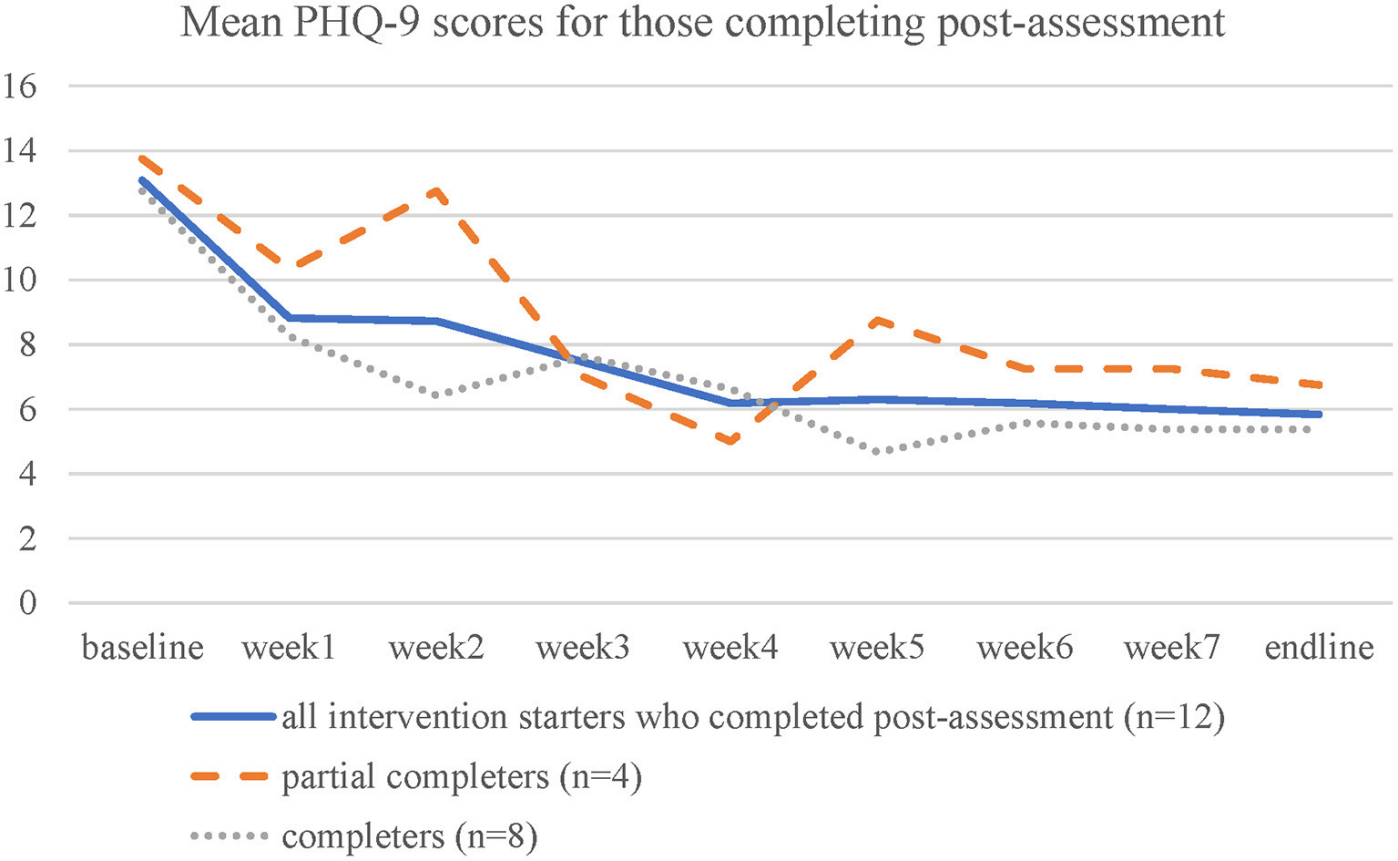
Mean PHQ-9 score in each week for participants who did some part of the intervention and completed post-assessment.

A significant decrease of PHQ-9 scores was observed [*t*_(11)_ = 4.29, *p* = 0.001, Cohen's *d* = 1.24, two-tailed]. Mean and *SD* of PHQ-9 scores at baseline and post-assessment among control, partial completers and completers are displayed in Table 4. Completers demonstrated the greatest reduction, followed by partial completers and control (see also Figures 3, 4).

**Table 4:** Means and SDs of clinical outcomes before and after intervention for control, partial completers and completers.

	**Control (*n* = 1)**		**Partial completers (3-4 sessions) (*n* = 4)**		**Full completers (5 sessions) (*n* = 8)**	
	**Pre**	**post**	**Pre**	**Post**	**Pre**	**post**
PHQ-9 (M, SD)	10	8	13.75 (4.5)	6.75 (4.65)	12.75 (2.38)	5.48 (6.61)
GAD-7 (M, SD)	8	12	11.25 (4.11)	7.25 (3.5)	9.00 (4.38)	6.50 (4.17)
WHO-5 (M, SD)	11	11	11.5 (1.0)	11.75 (5.32)	13.00 (7.84)	15.50 (5.90)
PSYCHLOPS (M, SD)	9	12	12.75 (4.5)	10.75 (3.5)	11.63 (5.90)	8.38 (4.24)

*PHQ-9, The Patient Health Questionnaire−9; GAD-7, The General Anxiety Disorder-7; WHO-5, The World Health Organization-Five Well-Being Index; PSYCHLOPS, the Psychological Outcome Profiles*.

### Secondary Outcomes

The complete case analysis showed a significant decrease of anxiety symptoms as measured with GAD-7 (*p* = 0.024, Cohen's *d* = 0.754, two-tailed) and self-defined problems as measured with PSYCHOLOPS (*p* = 0.005. Cohen's *d* = 0.99, two-tailed). A slight and non-statistically significant increase was observed for subjective well-being, *p* = 0.208, Cohen's *d* = 0.386, two-tailed.

### Qualitative Process Evaluation

Six participants were successfully contacted and willing to participate in the face-to-face qualitative interview.

#### Reach

Participants offered feedback and various suggestions to increase engagement and recruitment into subsequent trials. They agreed with the current strategy to recruit participants from within the residential college and the incentives we used. They suggested that utilizing social media channels (e.g., Facebook, Instagram, a WeChat official account), memes and other attractive marketing strategies, varying the content in simplified and traditional Chinese to attract the diverse student population, use of student groups, videos, and student email could increase recruitment. Additional feedback on the incentives was that bubble tea would be appropriate but stationary was not attractive. They also suggested that the use of a lucky draw that includes larger prizes can increase student engagement (e.g., tablet computers, video games consoles) in the recruitment.

#### Adoption

Adoption of intervention occurred at individual and organizational level. Participants suggested that the instructions were clear and the questionnaire length was appropriate, but the questions in the questionnaire are repeated (e.g., weekly assessment of depressive symptoms), so they may not have variation and the 2-weeks interval.

##### Impression

There were differences between students' initial impressions about the app and what they later came to realize about its function. For example, several students expected that this app would be boring, similar to other app programs they experienced. They were surprised and engaged by the content. Others considered it to be worthwhile, innovative, but not dynamic, as it did not respond to user inputs. Another student thought they would be speaking to a social worker, but then realized it was self-help.

##### Time to Use

Students varied with the time they used the app with several reporting late-night use, and others reporting use in the afternoon.

Students generally had positive feedback on the app. They considered it easy to use, with practical exercises, relatable stories, and engaging illustrated narrations, although a few students considered some scenarios to be less relevant or difficult to understand. Other concerns raised by the users about the app were that the audio exercise was too long that the user would get bored, app notifications were not frequent enough or occurred at an unwanted time.

Regarding organizational support, the SbS research team partnered with a residential college that was responsible for participants and e-helpers recruitment and the Student Counseling Section of the Student Affairs Office of the University that helped to train the e-helpers and provide regular supervision. The three parties worked closely during the feasibility study. Based on feedback from the interviewees, the recruitment activities were appropriate and sufficient and minimal guidance offered by e-helpers were helpful, which indicated organizational support was delivered well and led to the acceptance of recruitment strategies and guided support.

#### Effectiveness

Participants reported that they learned common stories about University students that remind them they were not alone, and further, they learned how to manage their emotions and schedule activities to help them relax and achieve their tasks. Activity scheduling, mood tracker, and relaxation exercise were most frequently used among the participants while a few participants found it difficult to master breathing exercises and were uncertain about what they could share in the gratitude exercise.

All participants received minimal support from e-helpers via a chatroom function in the app or phone calls, but not all participants availed themselves of this support because they did not encounter difficulties. For participants who were in need of support, they considered their e-helper helpful since they checked-in with them and listened to them. One participant reported that the e-helper prompted them to use the app and found this helpful.

Overall, participants were greatly satisfied with the program and the service they received, taking into account the function of the app, the improvement they perceived, and the knowledge or skills they acquired.

#### Implementation

Participants were satisfied with the SbS (range 7-8 out of 10, M = 7.5) regardless the number of completed sessions (range 3-5 sessions). Participants suggested a number of potential improvements to the program implementation. They suggested that the app could include videos as an alternative to illustrated narratives for those who preferred videos to text, and to add notifications when sessions were unlocked, accompanied by messages from their e-helper to keep themselves on track with the program, enhance notifications by making them visible similar to social networking applications, add additional timeslots for e-helpers to reply to user questions, add functions that facilitated interaction between the participant and characters and other study participants (e.g., group chat), add incentives within the app to encourage participants to engage in activities, and limit the amount of personal content requested by the app for the e-helper's monitoring.

All users universally reported a preference for female e-helpers for phone call support because of ease or willingness of disclosure of their problems to women.

#### Maintenance

Participants reflected several issues that should be addressed for the sustainability of the program. For instance, the app should avoid many open-ended questions in the questionnaire, provide sufficient information about the program (e.g., what participants are asked to do in the app) and sufficient notification to remind the participants to do the exercise or activities that they planned, offer timely responses by e-helpers, and provide additional assurances of confidentiality.

## Discussion

This feasibility study investigated the feasibility and acceptability of a culturally adapted, transdiagnostic mental health intervention to reduce depression among Chinese young adults. The study showed that the program was acceptable, feasible to implement, and promising given the reductions in symptoms observed among those who completed the intervention. Our study yielded a large effect size reduction in depressive symptoms and client-defined problem reduction and a medium effect size reduction of anxiety symptoms. Based on the qualitative feedback, the Chinese SbS showed a comparative satisfaction with another SbS program although a different evaluation was adopted (41). We also found a statistically non-significant trend of improvement in well-being. This study fills an important gap in the emerging evidence of the promise of app-based self-help within a Chinese population [for review, (8, 9)]. Notwithstanding the feasibility design and recruitment strategy of the current study, the results suggest that the mental health of University students, especially freshmen, was poor. Using a PHQ-9 cutoff score of 10 to identify potential users in a residential college of the University, the inclusion rate was about 22.0% These results also support the expansion of more accessible resources and support to this population (7, 32).

Attrition in digital interventions is high and varies across studies (44, 45). A meta-analysis (46) estimated that the average dropout rate of digital intervention for depression was nearly 50%. Due to the comparable participation rate with previous literature, we suggested that the Chinese SbS was feasible. Compared with Heim et al. (20) and Harper Shehadeh et al. (41), the non-starting rate (did not start any session) was 36.8% in this current study, which was lower than in other SbS studies (42.6 and 69.2%). Overall dropout rate (completed <4 therapeutic sessions) among those who started the intervention (*n* = 24) was 45.8% at post-assessment in this study, which was similar to other SbS studies at approximately 50%. People who did not start the program or dropped out from the study showed the greatest depressive and anxiety as well client-defined stress, compared with completers and starters at baseline (see Table 2). Their discontinuation may be due to the higher symptoms. The full program completion rate (completed all sessions) in this study was 37.5% (*n* = 9) among intervention starters, which was similar to Harper Shehadeh et al. (41) and Heim et al. (20), 43.2 and 32.8%, respectively. Completion rates (proportion of eligible participants who completed at least three therapeutic sessions in this study were comparable to other SbS studies, with 54.2% in the current study and 62.2% in (41) and 35.9 % in Heim et al. (20). The preliminary results showed that full completers showed the greatest improvement, and minimal difference in improvement between full completers (completed all sessions) and partial completers (completed 3-4 sessions) was observed (see Table 4), suggesting the treatment gains between the two groups of completers are comparable. However, it is worth mentioning that the partial completers seemed to have a higher level of symptoms than the full completers at baseline, which may impede their progress. Future studies could consider adjusting the guidance model to increase the participants' engagement in the digital program, which might lead to a better treatment outcome (47).

Attrition is typically greater in non-psychiatric samples and in interventions without real-person feedback and mood monitoring, compared with their counterparts (46). Although this is a common issue in digital mental health interventions, it can severely hinder the scalability and adoption of these interventions (1, 46). Two features of SbS, minimal guidance and mood monitoring, might play an important role to motivate the user to continue, assist users to overcome difficulties in completion of the intervention, and offer feedback and overview of user's mood over time (17, 48). In addition, the guidance model is critical in digital interventions for people with a higher level of depression as their greater improvement was associated with guided intervention while people with a subthreshold level of depression had similar gains with or without guidance during their intervention (47). These features were also universally mentioned and greatly accepted by the participants in the qualitative interview. Consistent with other adapted versions of the Step-by-Step program, the Chinese version showed preliminary evidence of significant improvement in depressive and anxiety symptoms, and client-defined stress (20, 41). Future studies that specifically target users who are seeking mental health support may yield greater completion rates.

Qualitative data provided insights into how the program can be improved. Participants suggested that study recruitment was sufficient as we adopted a variety of strategies to promote it within the residential college, including posters, mass email invitations, referrals, in-class recruitment, posts in social networking applications, and a booth in residential college events. It is recognized that self-stigma might hinder students from using the program (7). Based on previous work (49), recruitment strategies incorporated into this study attempted to use a stigma-free approach in promotion and recruitment to make the intervention or program more friendly and acceptable. Our study framed the program as a tool to help improve mood and academic stress, and most of the recruitment events were done in class, which was considered as stigma-free based on feedback from a participant because most of the students were doing the same task while another participant who participated in feedback session also reminded the recruitment team that outreach and recruitment should target students who need mental health support specifically.

Similar to Heim et al. (20), Chinese University students recommended more engaging activities and features in the app. Suggestions and recommendations from users were carefully considered before program refinement and the conduct of a definitive RCT (24). Changes made for the RCT included providing a download link for users who did not have an application download store on their device, adjusting the time used in the program for the time zone where the program was delivered and not too late, changing the topic selection in the chatroom to show up for the first message a user sent and would be deactivated for subsequent messages within a certain timeframe, offering notification for session unlock events, enabling control condition and randomization, providing a Chinese version of email reminders, increasing the number of seats in the app for RCT to allow more people to be screened, and changing the PHQ-9 cutoff used in screening to a score of 5 to reflect the range of distress in the population and to allow more participants to participate. Detailed qualitative information based on RE-AIM should be reported in the definitive RCT to thoroughly evaluate the entire program implementation process.

The current study has several limitations. Although a feasibility or pilot study is common practice and recommended for evaluation of recruitment potential, preliminary effect size and sample size estimations, and safety assessment before running the main trial, the results should be cautiously interpreted (50), and the effect sizes observed among completers may not be observed in the full trial. The current study design was an uncontrolled, single-arm pilot trial with a small sample size and not fully powered to detect significant changes over time. Also, the uncontrolled feasibility study was unable to inform the subsequent RCT regarding dropout and their reasons in the active control group, which is brief psychoeducation with a contact list of community services. Hence, future studies need to take a more conservative approach to estimate the actual sample size needed in the definitive RCT and take proper measures to reduce dropout in the control group. In addition, the current study piloted SbS in one residential college in one University, which limits generalizability. However, it fulfilled the purpose of the current study—to test the intervention and study procedure in order to inform the conduct of a definitive RCT. Our results revealed that a larger proportion of the participants were female, which might limit the interpretation of the results of this population. Indeed, Hall et al. (32) showed that females reported more mood symptoms than their male counterparts, and this, coupled with a higher proportion of women enrolled in University, can be plausible reasons more female students were included.

Despite the limitations, our study successfully piloted the intervention and the quantitative and qualitative data demonstrated the potential of the program to improve mental health among Chinese University students and provided information for the program amendment, while also supporting that a multi-site collaboration within the academic institution or a larger care setting was feasible. Establishing a strong partnership and collaboration with community stakeholders was beneficial to study implementation and future program sustainability.

## Data Availability Statement

The raw data supporting the conclusions of this article will be made available by the authors, without undue reservation.

## Ethics Statement

The studies involving human participants were reviewed and approved by University of Macau Research Ethics Board. The patients/participants provided their written informed consent to participate in this study.

## Author Contributions

BH conceptualized the project. BH and AL supervised the study. HS, AL, and BH conducted the study with the support of IH, SB, ES, and WC. HS organized the database, conducted qualitative interviews, and performed data analysis. BH and WC supervised data analysis. HS wrote the first draft of the manuscript. IH, SB, ES, MW, WC, AL, and BH carefully reviewed and edited the manuscript. All authors contributed to manuscript revision, read, and approved the submitted version.

## Funding

This work was funded by Macau Foundation and Macao SAR Government and RSTKO (PI: Hall) MYRG2018-00241-FSS.

## Conflict of Interest

The authors declare that the research was conducted in the absence of any commercial or financial relationships that could be construed as a potential conflict of interest.

## Publisher's Note

All claims expressed in this article are solely those of the authors and do not necessarily represent those of their affiliated organizations, or those of the publisher, the editors and the reviewers. Any product that may be evaluated in this article, or claim that may be made by its manufacturer, is not guaranteed or endorsed by the publisher.

## Abbreviations

CONSORT: Consolidated Standards of Reporting Trials
GAD-7: The General Anxiety Disorder-7
LMIC: low- and middle-income countries
PHQ-9: The Patient Health Questionnaire-9
PSYCHOLOPS: Psychological Outcome Profiles
SbS: Step-by-Step
WHO: World Health Organization
WHO-5: The World Health Organization-Five Well-Being Index.
