# Supplementary material for: A Feasibility Study of the WHO Digital Mental Health Intervention Step-by-Step to Address Depression Among Chinese Young Adults
Source: Front Psychiatry. 2022 Jan 7;12:812667. doi: 10.3389/fpsyt.2021.812667 (PMC8777229; doi:10.3389/fpsyt.2021.812667)
Supplement: Supplementary file 1 [file Data_Sheet_1.docx]

Supplementary Material

**Appendix 1: Qualitative Guide**

**Qualitative interview introduction**

- Self-introduction (your name, from CMS)
- Primary goal of the interview: Understand experience using the app, adherence to the program, challenges in using SbS, ways to improve, changes in mood or behavior
- Ask participant’s consent to be interviewed; confidentiality is guaranteed; mention compensation (voucher worth of MOP100 + post-assessment MOP100)
- Ask participant’s consent to be recorded, emphasize that the recording is only for evaluation and their personal information will not be revealed. If they refuse, more care will be needed to document call in the interview notes

**Informed consent**

- Written consent / verbal consent
- Obtained: Yes / No

**Background information**

1. Tell me about a typical day/ What is a typical day like for you?
   - What are the typical activities you have?
   - How often do you spend time with friends?
   - Who do you spend most of your free time with?
   - Where do you spend most of your free time?

**Study process**

1. When did you install/ use SbS for the first time?
2. How did you use it, did you install the app it directly on your phone or via the mobile website version?
   - Where did you get to know the program SbS?
3. What places/platforms would you suggest promoting the SbS?
4. How do you think about the current advertisement?
   - Do you think the advertisement is enough?
   - What kind of advertisement would be useful to attract students like you to use the app?
5. Did you complete all the assessments?
6. If yes, did you encounter any problems with completing assessment? If yes, what are they?
7. If not, what stopped you finishing them?

- How long did it take to complete the assessment?
- What do you think about the length of the assessment? Appropriate?

**SbS experience**

1. Did you use the traditional Chinese version of simplified Chinese version?
   - How was it? (ie easy/difficult to be understood?)？
2. What did you think about SbS before installing/ using it? After you installed/ used it?
   - Did the program match with your idea? (please elaborate)
3. When did usually you use the app (e.g., during the day, evening; weekday, weekend; etc) and for how long?
4. What did you like from the app (ask them to elaborate their answer)?
5. What have you learned from the program?/ What benefit did you receive from using SbS?
6. Did you use the features (ie Mood Tracker) in SbS [If not, what were some reasons they did not use these features?]*
7. Which of the exercises did you complete from the program?
8. Did you use any of these exercises/activities in your day to day life?
   - If yes, which ones? How was it?
9. Did you encounter any problems when using SbS? If yes, could you please elaborate?
10. Did you receive a call from e-helper? Did you pick it up? [If not, what were some reasons not to pick the call?]
    - What was your e-helper like?
    - Were they helpful?
11. [*if the user discontinued using the app*] Could you tell us some of the reasons you might have that hinder or make you difficult to continue the use of SbS?*
12. We really want to make SbS better for other Chinese students who use it. We can really use your help with this. I’d like to learn from you – how could it have been improved?
13. On a scale from 1-10, 10 being the best, how satisfied were you with this program to help you to reduce your stress?

**Close**

1. Thank the client for their time.
